# Hypoxia-inducible factor-1α regulates microglial functions affecting neuronal survival in the acute phase of ischemic stroke in mice

**DOI:** 10.18632/oncotarget.22851

**Published:** 2017-12-01

**Authors:** Seoyeon Bok, Young-Eun Kim, Youngsik Woo, Soeun Kim, Suk-Jo Kang, Yoontae Lee, Sang Ki Park, Irving L. Weissman, G-One Ahn

**Affiliations:** ^1^ Division of Integrative Biosciences and Biotechnology, Pohang University of Science and Technology (POSTECH), Pohang 37673, Korea; ^2^ Department of Life Sciences, Pohang University of Science and Technology (POSTECH), Pohang 37673, Korea; ^3^ Department of Biological Sciences, Korea Advanced Institute of Science and Technology (KAIST), Daejeon 34141, Korea; ^4^ Stem Cell Institute and Regenerative Medicine, Stanford University School of Medicine, Stanford, CA 94305, USA; ^5^ Current/Present address: Department of Radiation Oncology, Yonsei University College of Medicine, Yonsei University Health System, Seoul 03722, Korea

**Keywords:** hypoxia-inducible factor-1α (HIF-1α), microglia, stroke, phagocytosis

## Abstract

Cells universally adapt to ischemic conditions by turning on a transcription factor hypoxia-inducible factor (HIF), in which its role is known to differ widely across many different types of cells. Given that microglia have been reported as an essential mediator of neuroinflammation in many brain diseases, we examined the role of HIF in microglia in the progression of an acute phase of ischemic stroke by challenging our novel strains of myeloid-specific *Hif-1α* or *Hif-2α* knockout (KO) mice created by Cre-loxP system via middle cerebral artery occlusion (MCAO). We observed that *Hif-1α* but not *Hif-2α* KO mice exhibited an improved recovery compared to wild-type (WT) mice determined by behavioral tests. Immunostaining analyses revealed that there were increased numbers of both mature and immature neurons while microglia and apoptotic cells were significantly decreased in the dentate gyrus of *Hif-1α* KO mice following MCAO. By isolating microglia with fluorescence-activated cell sorter, we found that HIF-1α-deficient microglia were impaired in phagocytosis, reactive oxygen species (ROS) production, and tumor necrosis factor-α (TNF-α) secretion. We further observed a significant decrease in the expression of *Cd36* and *milk fat globule-epidermal growth factor 8* (*Mfg-e8*) genes, both of which contain hypoxia-responsive element (HRE). Knocking down either of these genes in BV2 microglial cells was sufficient to abrogate HIF-mediated increase in phagocytosis, production of intracellular ROS, or TNF-α secretion. Our results therefore suggest that HIF-1α in microglia is a novel therapeutic target to protect neuronal survival following an acute phase of ischemic stroke.

## INTRODUCTION

Ischemic stroke occurs by blood clots obstructing the blood flow to the brain leading to permanent neuronal disabilities in the affected individuals [1]. Cells respond to ischemic conditions by activating hypoxia-inducible factor (HIF), a transcription factor regulating numerous genes involved in cellular survival, migration, metabolism, and angiogenesis [2]. HIF is a heterodimer composed of an oxygen-sensitive α (HIF-α) subunit and oxygen-insensitive β subunit [2]. To date, three HIF-α isoforms (HIF-1α, HIF-2α, and HIF-3α) have been identified where HIF-α form is being hydroxylated under well-oxygenated conditions by proline hydroxylases, which eventually becomes degraded by proteasome via von Hippel Lindau E3 ubiquitin ligase complex [3]. Under hypoxic conditions, HIF-α cannot be hydroxylated due to the lack of oxygen inactivating proline hydroxylase activity [4]. HIF-α is then stabilized in the cytoplasm where it interacts with HIF-1β, followed by translocation into the nucleus where it binds to hypoxia-responsive elements (HREs) of HIF target genes [4].

Microglia are brain-resident macrophages rapidly responding to variety of stimuli in response to host-defense mechanisms protecting the brain against pathogens and injuries including ischemic stroke [5]. Upon activation, they are known to undergo morphological changes, upregulate markers including CD68 and major histocompatibility complex class II [6], and produce proinflammatory cytokines such as tumor necrosis factor-ɑ (TNF-ɑ) and interleukin-6 (IL-6) [6]. Activated microglia are commonly observed in many brain diseases such as stroke [7], Alzheimer’s disease [8], and Parkinson’s disease [9] and have recently been reported as a key mediator for neuroinflammation [10]. It is highly controversial whether activated microglia are detrimental or beneficial in the brain disease progression as some of recent studies have suggested that they are essential for protecting neurons by removing apoptotic cells and debris via phagocytic processes [11] or by trimming the neuronal synapses to regulate synaptic development [12].

It has been previously reported that microglia exposed to hypoxic conditions produce proinflammatory mediators such as nitric oxide (NO) [13] and TNF-ɑ [14] in a HIF-1-dependent mechanism. However, it is still poorly understood how HIF regulates microglial functions in ischemic stroke. Here we demonstrate by using our novel strain of myeloid-specific *Hif-ɑ* knockout (KO) mice that HIF-1α, but not HIF-2α in microglia critically affects neuronal survival in mice following ischemic stroke by regulating CD36 or milk fat globule-epidermal growth factor 8 (MFG-E8)-mediated phagocytosis, which in turn leads to reactive oxygen species (ROS) and TNF-α production. We therefore believe that HIF-1α in microglia may be a novel therapeutic target to promote neuronal survival in the hippocampus at the acute phase of ischemic stroke.

## RESULTS

### Myeloid-specific *Hif-1α* KO mice exhibit faster behavioral recovery following MCAO

**Figure 1:**
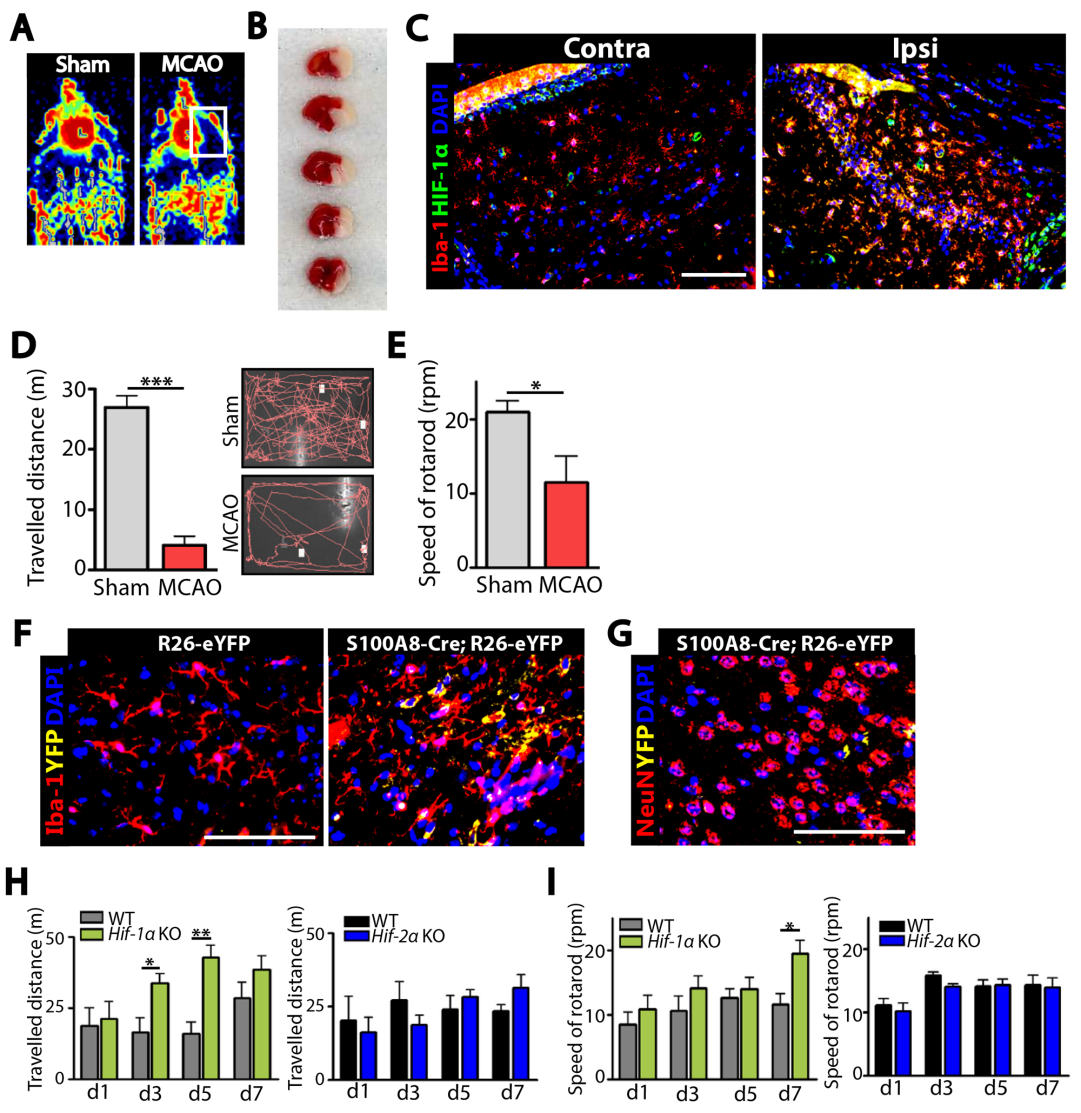
Stroke induction via middle cerebral artery occlusion (MCAO) demonstrates that our novel strain of myeloid-specific *Hif-1α* knockout (*Hif-1α* KO) mice exhibit an improved behavioral recovery than wild-type (WT) mice **(A)** Blood flow to the brain of wild-type (WT) mice during sham (left) or MCAO (right) surgery, as monitored by Laser Doppler Flowmetry. **(B)** Infarct regions (white) in the WT brain at 24 hr after MCAO stained with triphenyl tetrazolium tetrachloride. **(C)** Immunostaining of the WT brain at the contralateral (Contra; left) or ipsilateral (Ipsi; right) side at 24 hr post-MCAO for Iba-1 (red) to stain for microglia or HIF-1α (green). **(D)** Open-field behavioral test measuring exploratory behavior of WT mice subjected to sham or MCAO, measured for 10 min. **(E)** Rotarod test of mice WT subjected to sham or MCAO measuring the speed of rotarod at which mice fall. Data in D and E are the mean ± s.e.m. (n ≥ 5 mice for each group). **(F)** Immunofluorescence staining of the brain of Rosa26-eYFP reporter mice crossbred with Cre-hS1008 mice for Iba-1 (red). Note that only those mice bearing *Cre*-transgene (hS100A8-Cre;Rosa26-eYFP, right) demonstrated YFP (yellow) signal. **(G)** Immunofluorescent images of the Rosa26-eYFP reporter mice bearing *Cre*-transgene as in F stained using NeuN antibodies (red) to detect neuronal cells. For C, F, and G, nuclei are counterstained with DAPI (blue) and the scale bars denote 100 μm. **(H)** Open-field and **(I)** rotarod tests for WT (n ≥ 4), *Hif-1ɑ* KO (n = 5; green bars), or *Hif-2ɑ* KO (n = 5; blue bars) mice challenged with MCAO. Data in H and I are the mean ± s.e.m. with ^*^ and ^**^ indicate *P* < 0.05 and < 0.01, respectively, determined by Student’s *t*-test.

We first confirmed that MCAO resulted in an obstruction of blood flow (Figure 1A), development of infarcted region (Figure 1B), and an increased HIF-1α protein expression (Figure 1C) in the ipsilateral side of the brain. We further observed that mice subjected to MCAO exhibited a significant impairment in behavior as determined by open-field (Figure 1D) and rotarod (Figure 1E) tests. To determine whether our hS100A8 myeloid promoter targets microglia in the brain, we stained the brain of Rosa-eYFP reporter mice crossbred with Cre-hS100A8 mice. We observed eYFP-positive cells only in the brain of mice bearing *Cre*-recombinase gene (Figure 1F) and that these cells were highly co-localized with Iba-1-positive microglia (Figure 1F) but not with NeuN-positive neurons (Figure 1G). To determine a role of HIF-1 in microglia in ischemic stroke, we challenged our novel strain of myeloid-specific *Hif-a* KO mice (hereafter denoted as *Hif-α* KO mice) with MCAO. We found in *Hif-1α* KO mice that *Hif-1α* gene deletion efficiency was approximately 70% in Iba-1-positive microglia isolated by fluorescence-activated cell sorting (FACS) (Supplementary Figure 1A). Upon challenging *Hif-α* KO mice with MCAO, we observed that *Hif-1α* (Figure 1H and 1I) but not in *Hif-2α* KO mice (Figure 1H and 1I) exhibited a significantly faster behavioral recovery, as determined by open-field (Figure 1H) and rotarod (Figure 1I) tests compared to the wild-type (WT) control mice.

### Myeloid-specific *Hif-1α* KO mice have fewer infiltrating microglia and apoptotic neurons in the hippocampus following MCAO

**Figure 2:**
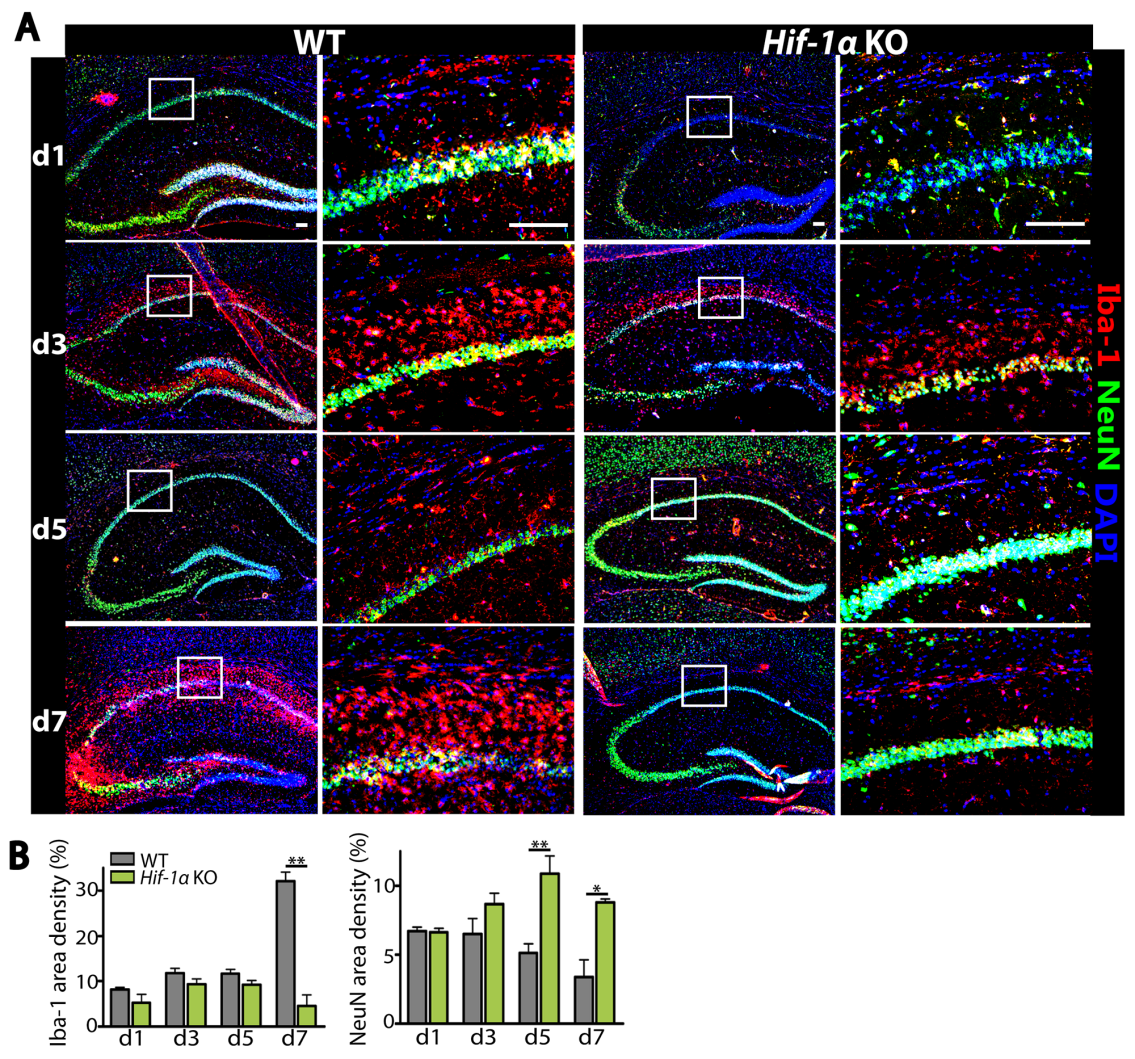
Decreased infiltrating microglia while increased number of neurons are observed in *Hif-1α* KO mice following MCAO **(A)** Immunostaining of the hippocampal areas of WT or *Hif-1α* KO mice following MCAO using Iba-1 (red) and NeuN (green) antibodies. Nuclei are counterstained with DAPI (blue). Scale bars denote 100 μm. **(B)** Quantification of results from A for Iba-1 (left) and NeuN (right). Data are the mean ± s.e.m. with ^*^ and ^**^ indicate *P* < 0.05 and < 0.01, respectively as determined by Student’s *t*-test.

**Figure 3:**
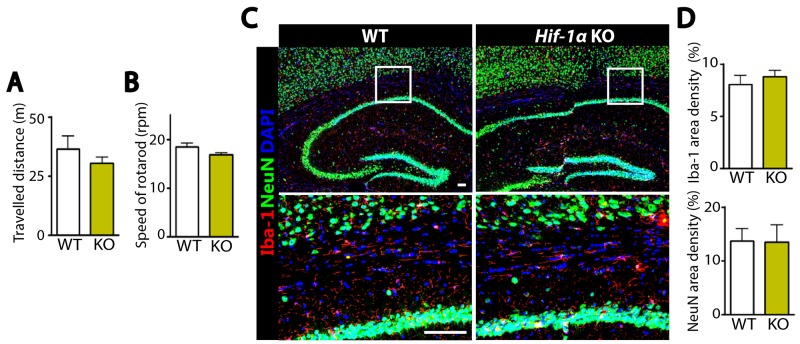
Without MCAO *Hif-1α* KO mice demonstrate similar behavioral outcome and comparable numbers of microglia and neurons to WT mice **(A)** Open-field and **(B)** rotarod tests in WT or *Hif-1α* KO mice not subjected to MCAO (n = 7 mice per group). **(C)** Immunofluorescence staining of the brains using Iba-1 (red) and NeuN (green) antibodies in WT or *Hif-1α* KO mice not challenged with MCAO. Nuclei are counterstained with DAPI shown in blue. Scale bars indicate 100 μm. **(D)** Quantification of Iba-1 (upper) or NeuN (lower) area densities in C. Data are the mean ± s.e.m. for at least three independent fields examined per mouse, n = 4 mice per group.

To determine how *Hif-1α* KO mice exhibited a faster recovery following MCAO, we examined microglia and neurons in the hippocampal areas of the ipsilateral side of the brain by immunostaining. We observed that while the numbers of Iba-1-positive microglia and NeuN-positive neurons were similar at d1 and d3 following MCAO between *Hif-1α* KO and WT mice (Figure 2), Iba-1-positive microglia were significantly fewer at d7 while NeuN-positive neurons were significantly increased at d5 and d7 in *Hif-1α* KO mice (Figure 2). To examine whether *Hif-1α* KO mice without MCAO would exhibit any defects in the numbers of neurons or microglia, we performed behavioral tests and immunostaining in *Hif-1α* KO or WT mice not challenged with MCAO. We found that behavioral outcome (Figure 3A and 3B) and the numbers of Iba-1-positive microglia and NeuN-positive neurons (Figure 3C and 3D) were all comparable between *Hif-1α* KO and WT mice.

**Figure 4:**
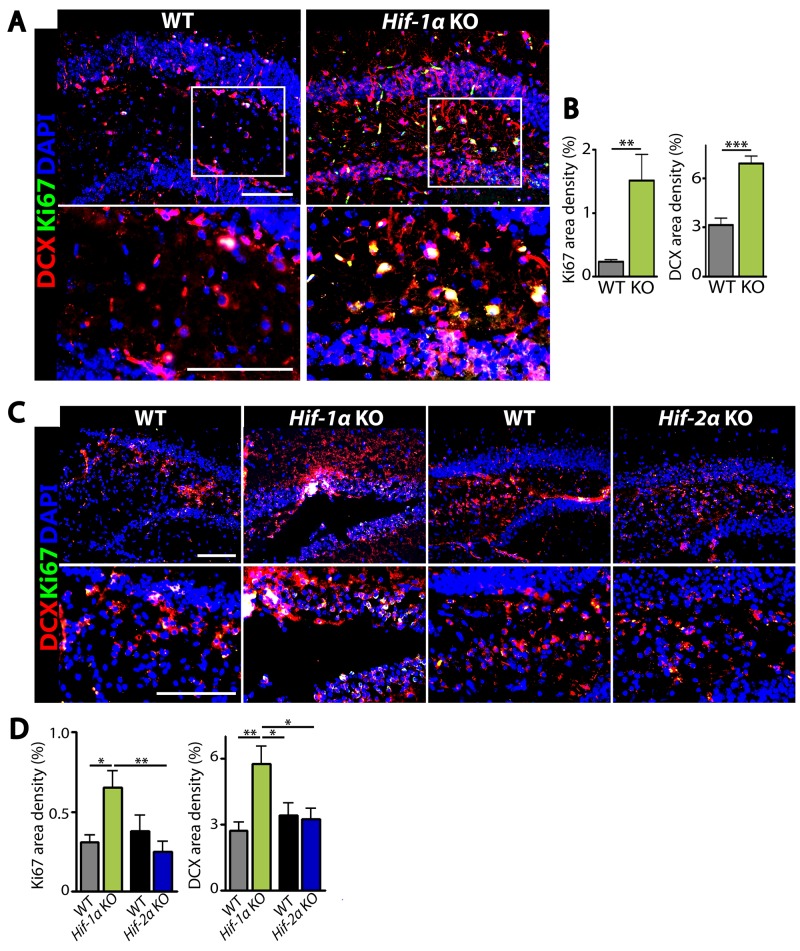
*Hif-1α* KO mice have increased proliferating immature neurons in the dentate gyrus of hippocampus after MCAO **(A)** Immunostaining images of dentate gyrus of the hippocampal areas in WT or *Hif-1α* KO mice at d5 post-MCAO for immature neurons and proliferating cells by using antibodies against DCX (red) and Ki67 (green), respectively. **(B)** Quantification of Ki67 (left) and DCX (right) area densities from A. **(C)** Immunofluorescent images of dentate gyrus regions in WT, *Hif-1α* KO or *Hif-2α* KO at d7 post-MCAO for proliferating neurons as in A. Nuclei are shown in blue and scale bars indicate 100 μm. **(D)** Quantification of Ki67 and DCX from C. Data in B and D are the mean ± s.e.m. for at least three independent fields examined per mouse, n ≥ 4 mice per group. ^*^, ^**^, and ^***^ denote *P* < 0.05, 0.01, and 0.001, respectively.

**Figure 5:**
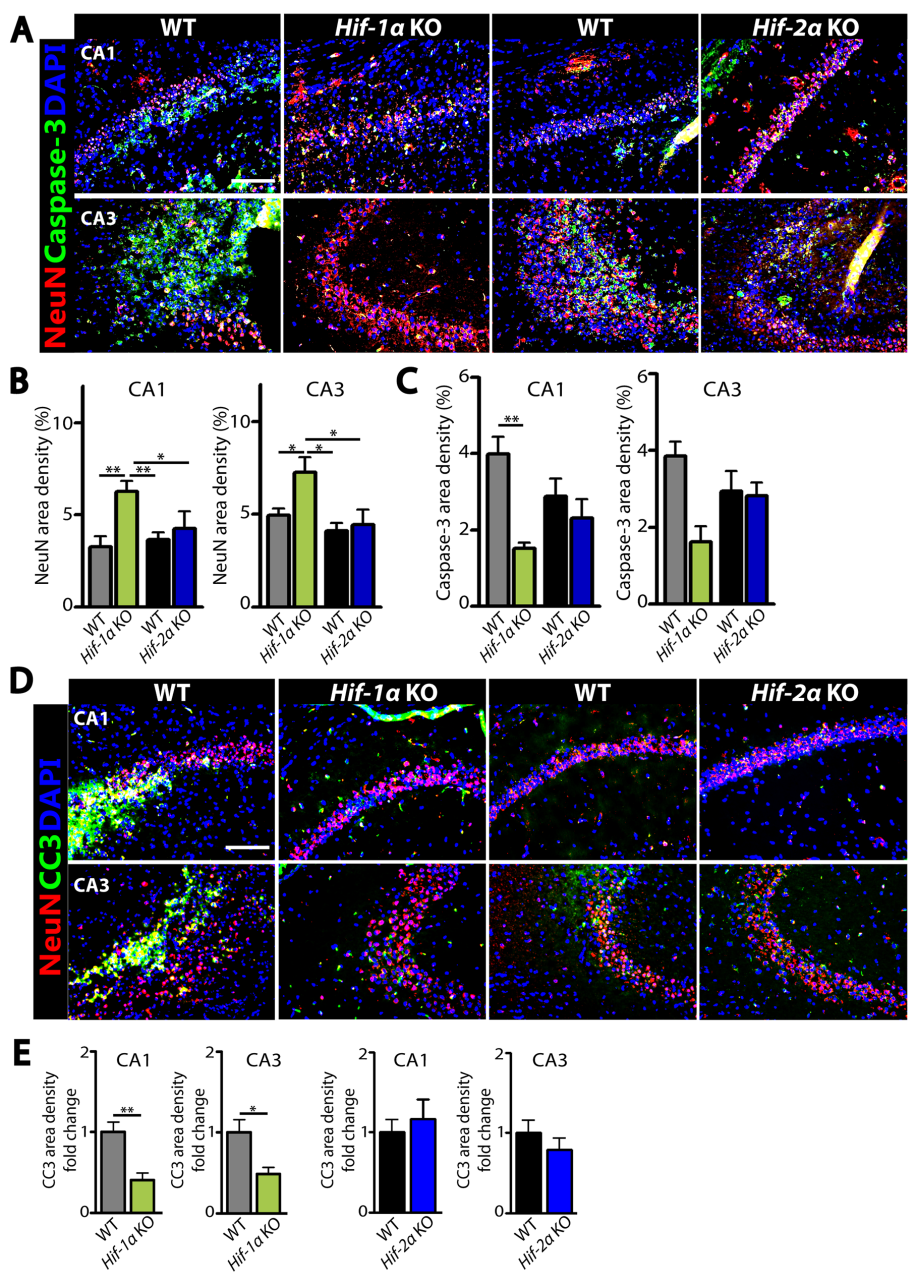
Decreased apoptotic neurons in the dentate gyrus of hippocampus in *Hif-1α* KO mice following MCAO Immunofluorescent staining images **(A**, **D)** and quantification of the staining results **(B**, **C**, and **E)** in the brain obtained from WT, *Hif-1α* KO or *Hif-2α* KO at d7 post-MCAO for neurons and apoptotic cells by using NeuN (red; A and D), caspase-3 (green; A), cleaved caspase-3 (CC3, green; D) antibodies. Nuclei are shown in blue with DAPI counterstain. The scale bars in A and D indicate 100 μm. Data in B, C, and E are the mean ± s.e.m. for at least three independent fields examined per mouse, n ≥ 4 mice per group. ^*^ and ^**^ indicate P < 0.05 and 0.01, respectively, determined by one-way ANOVA (B and C) or Student’s *t*-test (E).

The above results suggest that the increased number of neurons in *Hif-1α* KO mice following MCAO could be due to an increased level of cellular proliferation or a decreased level of apoptosis. To test these hypotheses, we performed immunostaining by using antibodies against Ki67 to detect proliferating cells, doublecortin (DCX) to stain newly-born immature neurons, or cleaved caspase-3 (CC3) to detect apoptotic cells in the dentate gyrus of hippocampus where adult neurogenesis in mice is known to predominantly occur [15]. We observed that the numbers of Ki67- or DCX-positive cells were significantly increased in *Hif-1α* KO mice than those in WT control at d5 (Figure 4A and 4B) and d7 (Figure 4C and 4D) post-MCAO. Furthermore, Ki67-positive cells were highly co-localized with DCX-positive cells in the granular layer in *Hif-1α* KO mice (Figure 4A and 4C), suggesting that *Hif-1α* KO mice have proliferating neuroblasts in the hippocampus following MCAO. In *Hif-2α* KO mice we observed similar numbers of Ki67- or DCX-positive cells compared to WT mice (Figure 4C and 4D). Immunostaining for neurons and apoptotic cells by using NeuN and caspase-3 or CC3 antibodies revealed that the numbers of neurons were increased (Figure 5A and 5B) while those of apoptotic cells were significantly decreased (Figures 5A, 5C–5E) in *Hif-1α* KO mice compared to those in WT control mice at d7 post-MCAO. We did not observe such differences in *Hif-2α* KO following MCAO (Figure 5).

### HIF-1α regulates microglial phagocytosis and abilities to produce ROS and TNF-α under ischemic conditions

**Figure 6:**
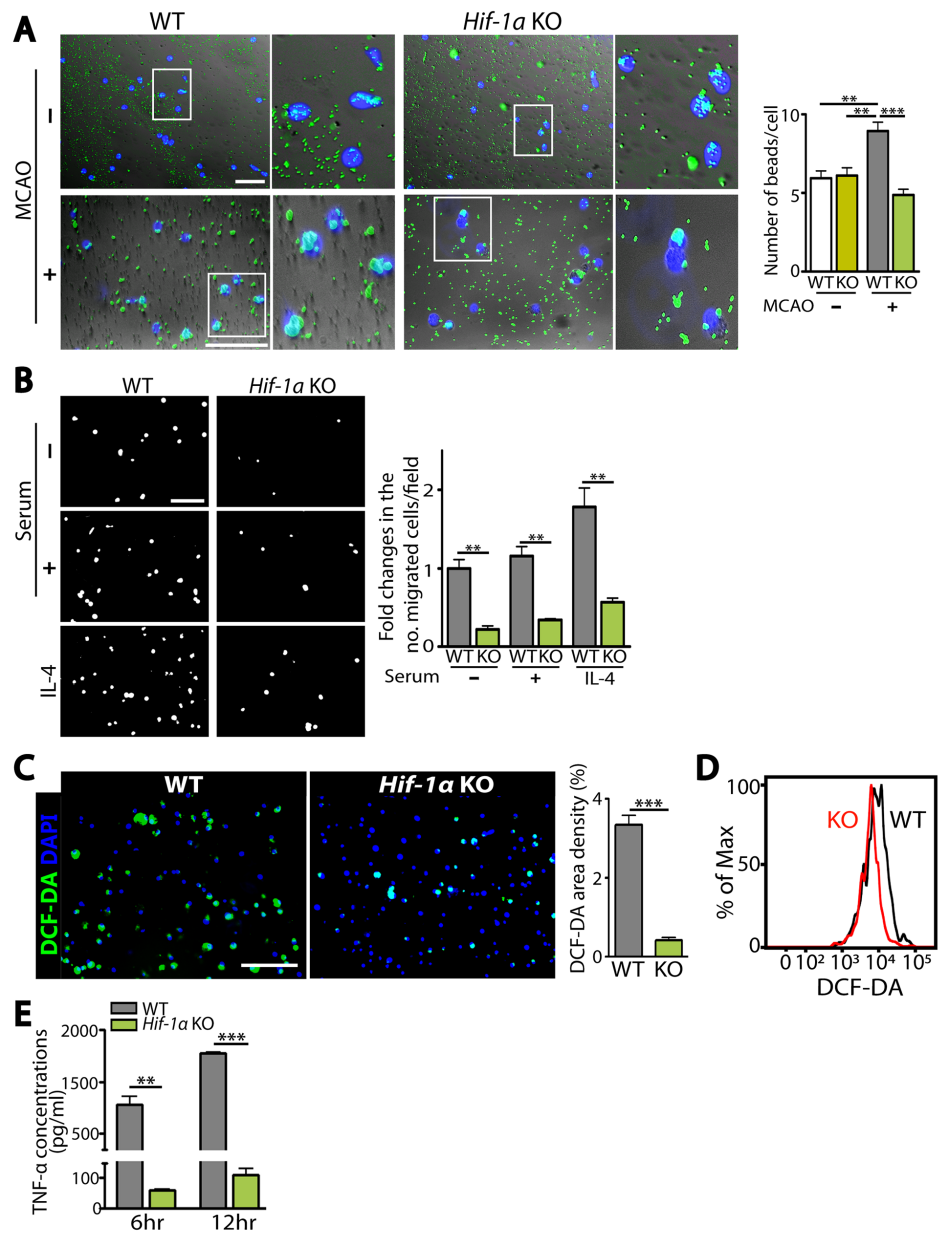
HIF-1α-deficient microglia are impaired in microglial functions including phagocytosis, chemotaxis, and production of ROS and TNF-α **(A)** Representative images (left) and quantification (right) of phagocytic uptake of fluorescent beads in microglia isolated from WT or *Hif-1α* KO mice at d5 post-MCAO. White boxes are magnified and shown on the right. **(B)** Representative images of transmigrated microglia, prepared as in A towards the lower compartment of modified Boyden chamber containing media supplemented with no serum, 10% serum, or IL-4. Quantification of the number of migrated microglia per field is shown in the bar graph. **(C)** Representative images of microglia prepared as in A incubated with DCF-DA. Quantification of FITC-positive area densities are shown in the bar graph. Nuclei in A and C are counterstained with DAPI (blue). Scale bars in A, B, and C indicate 100 μm. **(D)** Histogram analyses by FACS for DCF-DA fluorescence intensity in microglia from WT (black) or *Hif-1α* KO (red) mice, as prepared in A. **(E)** ELISA measurements of TNF-α concentrations at 6 and 12 hr in the supernatant obtained from microglia isolated as in A. Numbers in the bar graphs of A, B, C, and E are the mean ± s.e.m. with ^**^ and ^***^ indicate *P* < 0.01 and < 0.001, respectively.

We hypothesized that fewer infiltrating microglia and higher numbers of neurons in *Hif-1α* KO mice following MCAO were due to the deficiency of HIF-1α impairing microglial functions such as migration and phagocytosis. To test our hypothesis, we isolated Iba-1-positive microglia by FACS from WT or *Hif-1α* KO mice at d5 post-MCAO and examined microglial functions including phagocytosis, migration, ROS production, and TNF-α secretion. We observed that *Hif-1α*-deficient microglia exhibited significantly impaired phagocytic uptake of fluorescent beads compared to WT microglia (Figure 6A). This impaired phagocytosis was not observed in microglia isolated from *Hif-1α* KO mice not subjected to MCAO (Figure 6A), suggesting that HIF-1α regulates phagocytic activities in microglia selectively under hypoxic conditions. Phagocytic activation is also known to regulate other functions of macrophages/microglia such as chemotaxis [16] and production of cytokines or ROS [17]. Upon examining chemotaxis, intracellular ROS levels, and TNF-α concentrations in FACS-isolated microglia, we observed that HIF-1α-deficient microglia exhibited significant impairments in chemotaxis (Figure 6B), and in production of ROS (Figure 6C and 6D) and TNF-α (Figure 6E) compared to WT microglia.

### *Cd36* or *Mfg-e8* phagocytic gene mediates HIF-1α-regulated microglial functions

**Figure 7:**
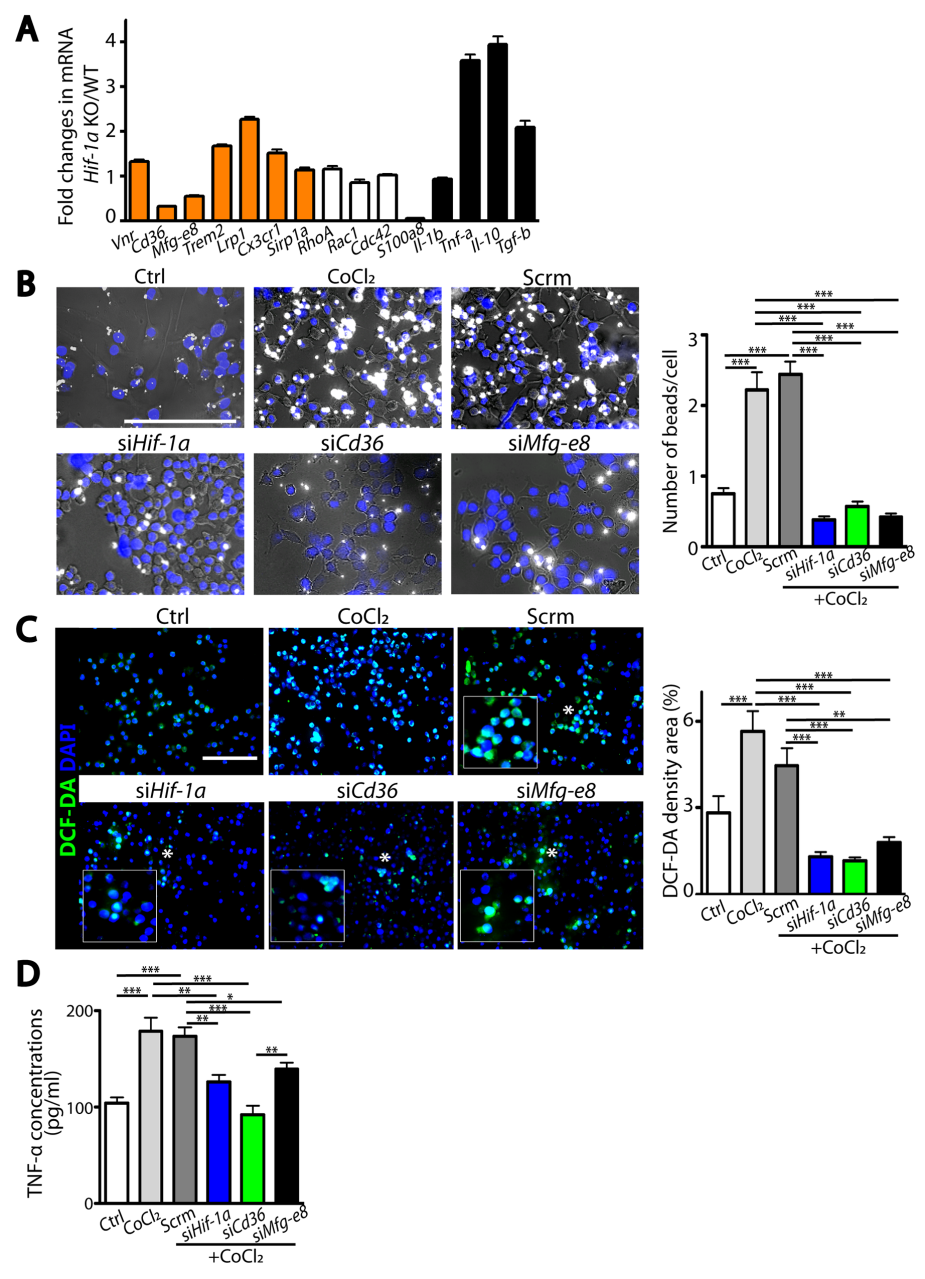
HIF-1α-regulated microglial functions are mediated by *Cd36* or *Mfg-e8* **(A)** Fold changes in mRNA in primary microglia isolated from *Hif-1α* KO mice compared to those in WT at d5 post-MCAO in pathways including phagocytosis, cell migration, and immune responses. **(B)** Representative images of phagocytic uptake of fluorescent beads (white) in BV2 microglial cells. Quantification of the number of phagocytosed beads per cell is shown on the right. **(C)** Representative images of BV2 cells incubated with DCF-DA (left) and quantification (right). White asterisks in the immunofluorescent images indicate magnified regions shown in the inserted white boxes. **(D)** TNF-α levels in the supernatant obtained from BV2 cells with or without transfection. Nuclei in B and C are counterstained by DAPI (blue) and scale bars denote 100 μm. Data from A to D are the mean ± s.e.m. for triplicate determinations with ^*^, ^**^, and ^***^ indicate *P* <0.05, <0.01, and < 0.001, respectively.

Because HIF-1 possesses numerous downstream target genes, we next sought how HIF-1α regulates microglial functions by examining gene expression changes in microglia isolated from *Hif-1α* KO or WT mice at d5 post-MCAO against a panel of pathways involved in phagocytosis, chemotaxis, and inflammatory responses. We observed that *Cd36* and *Mfg-e8* phagocytic gene expressions were significantly decreased in HIF-1α-deficient microglia (Figure 7A), suggesting that they may be HIF-1-regulated genes. Consistent with this, *Cd36* and *Mfg-e8* contained 5’-RCGTG-3’ putative hypoxia-responsive element (HRE) binding sites [18] at 10 kb upstream of transcription start sites (Supplementary Figure 1B), indicating that they are downstream targets of HIF. To determine whether *Cd36* or *Mfg-e8* can regulate HIF-1α-mediated microglial functions, we silenced *Hif-1α, Cd36* or *Mfg-e8* in BV2 microglial cell lines. We observed that phagocytosis (Figure 7B) and production of ROS (Figure 7C) and TNF*-*α (Figure 7D) were significantly increased by treatment with CoCl_2_, a HIF mimetic. Silencing *Hif-1α* effectively decreased HIF-1α protein levels (Supplementary Figure 1C) and *Cd36* or *Mfg-e8* gene expression only in CoCl_2_-treated conditions (Supplementary Figure 1D). We found that knocking down either *Cd36* or *Mfg-e8* could abrogate HIF-mediated increase in phagocytosis (Supplementary Figure 1B) and production of ROS (Supplementary Figure 1C) or TNF*-*α (Supplementary Figure 1D) in BV2 cells to the level comparable to *Hif-1α* siRNA (Supplementary Figure 1B–1D). These results suggest that phagocytic molecules CD36 and MFG-E8 mediate HIF-1α-regulated microglial functions.

## DISCUSSION

In this study, we report our findings where we observed a significantly faster behavioural recovery in our myeloid-specific *Hif-1ɑ* KO mice following ischemic stroke challenge. We demonstrate that these mice are impaired in HIF-1α-dependent microglial phagocytic functions mediated by *Cd36* and *Mfg-e8*, which can further regulate other functions such as ROS and TNF-α production. It has been previously reported that microglial expression of CD36, a highly glycosylated class B scavenger receptor, steadily increases during the acute phase (d3 and d7) of MCAO [19] and that inhibition of CD36 not only impairs phagocytosis but also attenuates damages and inflammatory responses mediated by cerebral ischemia, leading to better neurological functions [20, 21], consistent with our results. MFG-E8, also known as lactaherin, is a secretory glycoprotein that forms bridges between microglial receptors hence linking phosphatidylserine-exposing neurons to ɑ_V_β_3_ and ɑ_V_β_V_ integrins of phagocytic cells [22]. Effects of MFG-E8 on ischemic stroke is somewhat controversial. A study by Deroide and colleagues [22] have reported a larger infarct size in *Mfg-e8* KO mice at d7 following MCA electrocoagulation through a mechanism of inhibiting integrin-mediated IL-1β production. On the other hand, Neher and colleagues [23] have demonstrated a significantly reduced infarct size and improved motor functional recovery in *Mfg-e8* KO mice following intracerebral injection of endothelin-1. They have proposed that delayed phagocytosis of those glutamate-stressed, phosphatidylserine-exposed neuronal cells by microglia may be critical in preserve neuronal functions in mice [23].

It is highly controversial whether microglia play a beneficial or detrimental role in ischemic stroke. While some studies have demonstrated that depletion of microglia themselves can lead to an increased neuronal injury [24], others have reported neuronal protective effects [25]. The complex function of microglia becomes even more sophisticated by microenvironmental factors such as hypoxia and HIF, the major transcription factor activated under hypoxic condition. Although previous seminal study by Cramer and colleagues [26] has demonstrated that HIF-1α itself has shown a minimal effect on bacterial phagocytosis in ‘macrophages’, our results demonstrated that HIF-1α, but not HIF-2α exerts a major functional regulation in ‘microglia’. It is highly likely that different myeloid promoters (i.e., LysM *versus* hS00A8) utilized to create myeloid-specific *Hif-α* KO mice may target different subpopulations of myeloid cells. Indeed, we have recently reported that the angiogenic phenotype that we have observed in our myeloid-specific KO mice of constitutive HIF activation (by *von Hippel Lindau* deletion) was not observed when we created them using LysM promoter [27]. A recent study by Ko and colleagues [28] has utilized myeloid-specific *Hif-1ɑ* KO mice of LysM promoter and reported that these mice have also exhibited improved neurological functions following ischemia challenge in a mechanism by which HIF-1α-deficient microglia recruits less neutrophil infiltration into the infarcted regions.

In summary, we report that HIF-1α in microglia after stroke may facilitate phagocytosis and other microglial functions including chemotaxis, ROS production, and TNF-ɑ production through CD36 and/or MFG-E8 hence interfering with adult neurogenesis in the acute phase of ischemic stroke. Thus, our findings suggest that HIF-1α in microglia may be a potential therapeutic target for sparing neuronal loss and reducing neuroinflammation, which can lead to improved functional recovery in stroke patients.

## MATERIALS AND METHODS

### Mice

Cre-mediated inactivation of *Hif-1α* or *Hif-2α* in myeloid cells were accomplished by cross-breeding mice having lox-P flanking alleles in HIF-1α (*Hif-1α*^fl/fl^, The Jackson Laboratory) or HIF-2α (*Hif-2α*^fl/fl^, The Jackson Laboratory) with transgenic mice bearing the *Cre-recombinase* gene under the hS100A8 promoter (*Cre*-hS100A8) obtained from Dr. I. L. Weissman at Stanford University, as described previously [27]. *Cre*-hS100A8 mice were also crossbred with transgenic mice bearing lox-P-STOP-eYFP-lox-P under the ubiquitous Rosa26 promoter (Rosa26-eYFP) obtained from Dr. S.-J. Kang at Korea Advanced Institute of Science and Technology (KAIST). Mice were maintained in a germ-free environment and had access to food and water *ad libitum*. All animal procedures were approved by the Institutional Animal Care and Use Committee at Pohang University of Science and Technology.

### Cell culture conditions

Murine BV2 microglial cell line was kindly provided by Dr. Kyoungho Suk at Kyungpook National University, South Korea. BV2 cells were cultured in Dulbecco’s modified Eagle’s medium (DMEM) (Welgene) supplemented with 10% heat-inactivated fetal bovine serum (FBS) (Omega Scientific Inc), and 100 U/mL penicillin and 100 μg/mL streptomycin (Thermo Fisher Scientific).

### Middle cerebral artery occlusion (MCAO)

MCAO procedures were carried out as described previously [29]. Briefly, a silicon suture (Doccol Corporation) was used to occlude perfusion in the left MCA for 60 min, followed by removal of the suture and terminal ligation of the left carotid artery. Sham animals were similarly ligated for the left carotid artery. Blood perfusion before or during occlusion was measured by using Laser Doppler (PIM3, PERIMED) in the anesthetized mice that had been pre-warmed to the core body temperature of 38 °C on a heating plate measured by a rectal temperature probe (Physitemp).

### Triphenyltetrazolium chloride staining

The brain of euthanized animal was removed and transferred to an acrylic brain matrices (Leica Biosystems) and sectioned at 2 mm thickness from the frontal to the occipital pole. The slices were then immersed in 2% 2,3,5-triphenyltetrazolium chloride (Sigma) in phosphate buffered saline (PBS) and further incubated at room temperature for 15 min, followed by fixation with 4% paraformaldehyde (PFA) (DaeJung Chemicals) for 30 min. The brain slices were placed on transparent films and scanned with EPSON perfection V700 PHOTO scanner.

### Behavioral tests

Open-field test was performed based on the apparatus and procedure used by Crusio and Schweglar [30] with modifications. Briefly, mice were placed in an open box allowing to move freely for 10 min. Location and accumulated travelled distance for each mouse were recorded by a closed circuit digital camera and analyzed by using SMART software (Panlab). Rotarod test (Panlab) was performed with the rod of 50 mm in the width and 30 mm in the diameter. Mice were positioned on the rod heading in the opposite direction to the rotation. In a test session, mice were placed on the rods set to accelerate from 4 to 40 rpm during 5 min. Test runs were repeated for five times per day and the mean rotational velocities were recorded at the time of the fall.

### Immunostaining

Mice were cardiac perfused with 4% PFA (Daejung Chemicals) in PBS and the brain was harvested and made into frozen sections, followed by fixation using ice-cold 100% methanol for 10 min at the room temperature. Sections were then incubated with antibodies against Iba-1 (goat anti-mouse Iba-1 polyclonal antibodies, Abcam), CD68 (rat anti-mouse CD68 monoclonal antibodies, Abcam), HIF-1α (goat anti-rabbit HIF-1*α* antibodies, Novus), NeuN (rabbit anti-mouse NeuN antibodies, Abcam), DCX (rabbit anti-mouse DCX antibodies), Ki67-FITC (Biolegends), caspase-3 (rabbit anti-mouse antibodies, Abcam), or cleaved caspase-3 (CC3, rabbit anti-mouse CC3 antibodies, Cell signaling technology) antibodies for overnight at 4°C. Secondary antibodies of species-matched IgG conjugated with Alexa 488 and/or Alexa 546 (Thermo Fisher Scientific) were incubated for 1 hr at the room temperature. Sections were finally mounted with ProLong Gold antifade reagent with DAPI (Invitrogen) and examined with Zeiss Axio Scope with EC PLAN NEOFLUAR at 10×, 20×, and 40× objective lenses. Digital images were taken using AxioCam HRM camera and processed with AxioVision 4.8 software using 20× objective fluorescence microscope as described above. Images were evaluated at least three independent areas per mouse. Area densities were calculated by Image J software (National Institutes of Health).

### Fluorescence-activated cell sorting (FACS)

FACS procedures for microglia were performed as previously described [31]. In brief, left hemisphere including the infarct region of the brain was harvested from WT or *Hif-1α* KO mice and digested in an enzyme cocktail followed by introduction to Percoll (GE Healthcare) gradient (30%, 37%, and 70% Stock Isotonic Percoll balanced with Hanks balanced salt solution (Invitrogen). Microglia enriched at 70% - 37% interphase were then collected and stained with Iba-1 antibodies as described in the ‘immunostaining’ procedure. Cells were finally resuspended in PBS + 3% FBS (Invitrogen) containing propidium iodide and analyzed by BD LSR II (BD Biosciences) or sorted by MoFlo XPD (Beckman Coulter).

### Phagocytosis assay

BV2 murine microglial cell line or purified primary microglia by FACS were plated at a density of 5 × 10^4^ and 2 × 10^5^ cells, respectively, on poly-D-lysine-coated 8 chamber polystyrene vessel tissue culture treated glass slide (BD Falcon). Cells were left to adhere overnight in DMEM at 37 °C under 20% O_2_ with 5% CO_2_ followed by stimulation with 100 ng/mL LPS (Calbiochem), 50 ng/mL IFN-γ (Peprotech), 20 ng/mL IL-4 (Peprotech), or 150 μM CoCl_2_ (Sigma) for 6 hr. 3 μL of 1:10 diluted (1.1 × 10^9^ beads) 1 μm carboxylate-modified fluorescent microspheres (Invitrogen) were added and incubated for another 2 hr at 37 °C under 20% O_2_ with 5% CO_2_. Following incubation, medium was removed and ice-cold PBS was added to arrest the bead uptake. Cells were then fixed with 4% PFA, chambers were removed, and slides were mounted with Prolong Gold antifade reagent and analyzed by fluorescence microscopy as described above. The number of beads ingested per cell were counted for 100 cells from at least three independent experiments.

### Quantitative real-time polymerase chain reaction (qRT-PCR)

Genomic DNA was purified by using the PureLink Genomic DNA kit (Bioneer) from microglia sorted by FACS. Gene deletion efficiency was determined by qRT-PCR (OneStepPlus; Applied Biosystems) using the following primers: *Hif-1α* Fwd 5′-GGT GCT GGT GTC CAA AAT GTA G-3′ and *Hif-1α* Rev 5′ ATG GGT CTA GAG AGA TAG CTC CAC A -3′, *β-actin* Fwd 5′-AGA GGG AAA TCG TGC GTG AC-3′, *β-actin* Rev 5′-CAA TAG TGA CCT GGC CGT-3′. Genomic DNA level was normalized to *β-actin*. Total mRNA was isolated from FACS-purified microglia using RNeasy mini kit (QIAGEN) according to the manufacturer’s protocol. cDNA was synthesized using the following reagents: RNase-free DNase I (Promega), SUPERasein (Ambion), EDTA (Promega), dNTP (Invitrogen), random primers (Invitrogen), and Reverse Transcriptase (Promega). Synthesized cDNA was then subjected to PCR amplification using SYBR GREEN (Applied Biosystems) with primers listed below. mRNA levels were calculated by relative quantification using comparative threshold cycle values based on those of β-actin according to the manufacturer’s instructions (Applied Biosystems). Primer sequences were: *Cdc42*, 5’- TAC TGC AGG GCA AGA GGA TT -3’ and 5’- GTC CCA ACA AGC AAG AAA GG -3’; *Cd36*, 5’-GTC CTG GCT GTG TTT GGA -3’ and 5’- GCT CAA AGA TGG CTC CAT TG -3’; *Cx3cr1*, 5’- CAC CAT TAG TCT GGG CGT CT -3’ and 5’- GAT GCG GAA GTA GCA AAA GC -3’; *Hif-1a*, 5’- CAA GAT CTC GGC GAA GCA A -3’ and 5’- GGT GAG CCT CAT AAC AGA AGC TTT -3’; *Hif-2a*, 5’- CAA CCT GCA GCC TCA GTG TAT C -3’ and 5’- CAC CAC GTC GTT CTT CTC GAT -3’; *hS100A8*, 5’- CCA ATT CTC TGA ACA AGT TTT CG -3’ and 5’- TCA CCA TGC CCT CTA CAA GA -3’; *Il-1β*, 5’- GAG AAC CAA GCA ACG ACA AAA TAC C -3’ and 5’- GCA TTA GAA ACA GTC CAG CCC ATA C -3’; *Il-10*, 5’- GAT GCC CCA GGC AGA GAA -3’ and 5’- CAC CCA GGG AAT TCA AAT GC -3’; *Lrp1*, 5’- GAC AGC AAA CGA GGC CTA AG -3’ and 5’- ACA GGG GTT GGT CAC TTC AG -3’; *Mfg-e8*, 5’- TTC TGT GAC TCC AGC CTG TG -3’ and 5’- TGG CAG ATG TAT TCG GTG AA -3’; *Rac1*, 5’- TAT GGG ACA CAG CTG GAC AA -3’ and 5’- ACA GTG GTG TCG CAC TTC AG -3’; *Rhoa*, 5’- TGG TTG GGA ACA AGA AGG AC -3’ and 5’- ACA AGA TGA GGC ACC CAG AC -3’; *Sirp1α*, 5’- TCA GTA ATG TCA CCC CAG CA -3’ and 5’- ACC CCT TGG CTT TCT TCT GT -3’; *Tgfβ*, 5’- TGG AGC AAC ATG TGG AAC TC -3’ and 5’- CAG CAG CCG GTT ACC AAG -3’; *Tnfa*, 5’- CGA GTG ACA AGC CTG TAG CC -3’ and 5’- GGT TGA CTT TCT CCT GGT ATG AG -3’; *Trem2*, 5’- TAT GAC GCC TTG AAG CAC TG -3’ and 5’- AGA GTG ATG GTG ACG GTT CC -3’; *Vnr*, 5’- GAT GGC TGC GTA TTT TGG AT -3’ and 5’- TGG AAG TCT CCC ACT GCT CT -3’.

### TNF-ɑ ELISA

TNF-ɑ levels were measured by ELISA at 6 and 12 hr in the supernatants of primary microglia isolated from WT or *Hif-1α* KO mice at d5 post-MCAO or from BV2 cells by using mouse TNF-ɑ Quantikine ELISA System (R&D Systems) according to the manufacturer’s protocol.

### ROS production

DCF-DA (2’,7’-dichlorodihydrofluorescein diacetate; Molecular Probes) was used to study the intracellular ROS production in BV2 cells and primary microglia. The study was performed on poly-D-lysine-coated 8 chamber polystyrene vessel tissue culture treated glass slide (BD Falcon), wherein the cells were seeded at a density of 1 × 10^5^ cells in 500 μl of 10% FBS supplemented medium at 37 °C under 20% O_2_ with 5% CO_2_. After 4 hr of cell attachment, DCF-DA was added at a concentration of 10 μM in fresh media 500 μl/well and incubated for 30 min. Following incubation, the dye solution was removed and the cells were washed twice with 500 μl/well in PBS. DCF-fluorescence was determined by fluorescence microscopy and flow cytometry.

### siRNA transfection

Murine *Hif-1α*, *Cd36*, and *Mfg-e8* siRNA were purchased from Dharmacon and scramble siRNA from Bioneer. siRNA sequences were as follows: mouse *Hif-1α* siRNA, 5’-GGA AAG AGA GUC AUA GAA C-3’; mouse *Cd36* siRNA #1 Sense, 5’-CUG AGU AGG UUU UUC UCU U(dTdT)- 3’ and Antisense, 5’-AAG AGA AAA ACC UAC UCA G(dTdT)-3’; mouse *Cd36* siRNA #2 Sense, 5’-AGU CAU CAA UGU UCC UAC A(dTdT)-3’, Antisense, 5’-UGU AGG AAC AUU GAU GAC U(dTdT)-3’; mouse *Mfg-e8* siRNA #1 Sense. 5’-GAC UGU AUA UGA GGA GCA A(dTdT)-3’, Antisense, 5’-UUG CUC CUC AUA UAC AGU C(dTdT)-3’; mouse *Mfg-e8* siRNA #2 Sense, 5’-CAG UAU GUG GAG UCC UAC A(dTdT)-3’, Antisense, 5’-UGU AGG ACU CCA CAU ACU G(dTdT)-3’; Scramble siRNA, 5’-AUCCGCGCGAUAGUACGUATT-3’. siRNA transfection was performed with Lipofectamine RNAiMAX (Invitrogen). In brief, 60 pmol of siRNA were mixed with 10 μl of Lipofectamine RNAiMAX in 1ml of pure DMEM medium (Welgene). The mixture was added to BV2 cells that were 70% confluent in 6-well culture dishes without antibiotics. 24 hr after transfection, cells were then resuspended in complete DMEM medium, incubated for another 24 hr, and used for experiments.

### Western blot analysis

Cells were lysed in radioimmunoprecipitation assay (RIPA) buffer containing Protease Inhibitor Cocktail (Calbiochem). Nuclear and cytoplasmic fractions were prepared from the BV2 cells using NE-PER reagents [32] according to the manufacturer’s protocol. Protein concentrations were determined by BCA assay [32]. Samples were loaded on 12% Bis-Tris pre-cast polyacrylamide gel (Invitrogen) and transferred to PVDF membrane (BIO-RAD). Membrane was then probed with antibody against HIF-1ɑ (NB100-449, Novus) followed by goat anti-rabbit IgG antibody conjugated with horseradish peroxidase (Santa Cruz) and developed with the Pierce ECL substrate (Thermo Fisher Scientific).

### Statistical analysis

Statistical comparisons of the datasets were performed by unpaired, two-tailed Student’s *t* test, or one- or two-way ANOVA using Prism software (Version 4.00; GraphPad Inc.). Data were considered statistically significant when *P* < 0.05.

## SUPPLEMENTARY MATERIALS FIGURE



## Abbreviations

HIF: hypoxia-inducible factor
KO: knockout
WT: wild-type
MCAO: middle cerebral artery occlusion
ROS: reactive oxygen species
TNF-α: tumor necrosis factor-α
CD36: cluster of differentiation 36
MFG-E8: milk fat globule-epidermal growth factor 8
HRE: hypoxia-responsive element
IL-6: interleukin-6
DCX: doublecortin
eYFP: enhanced yellow fluorescent protein
CC3: cleaved caspase-3
DCF-DA: 2’,7’-dichlorodihydrofluorescein diacetate
ELISA: enzyme-linked immunosorbent assay
FACS: fluorescence-activated cell sorting
