# Supplementary material for: Hypoxia-inducible factor-1α regulates microglial functions affecting neuronal survival in the acute phase of ischemic stroke in mice
Source: Oncotarget. 2017 Dec 1;8(67):111508–21. doi: 10.18632/oncotarget.22851 (PMC5762339; doi:10.18632/oncotarget.22851)
Supplement: Supplementary file 1 [file oncotarget-08-111508-s001.pdf]

Hypoxia-inducible factor-1α regulates microglial functions affecting neuronal survival in the acute phase of ischemic stroke in mice

SUPPLEMENTARY MATERIALS

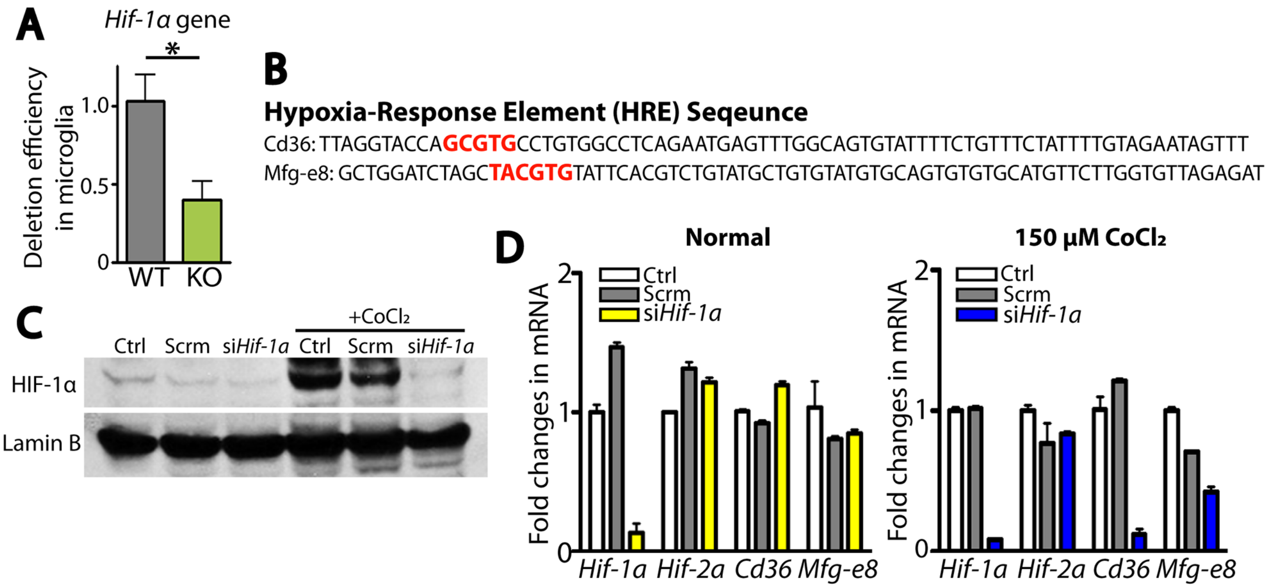

Supplementary Figure 1: (A) Deletion efficiency of *Hif-1α* measured in isolated microglia from WT or myeloid-specific *Hif-1α* KO mice at d5 post MCAO. **(B)** Hypoxia-responsive element (HRE) sequence, 5'-RCGTG-3' (red) in murine *Cd36* or *Mfg-e8*. **(C)** Immunoblot analyses against HIF-1α protein in the nucleus of BV2 cells knocked down for *Hif-1α* with and without $CoCl_2$. Lamin B was used as loading control. **(D)** Knockdown efficacy of *Hif-1α*, *Cd36,* and *Mfg-e8* in BV2 cells transfected with scrambled siRNA (Scrm), *Hif-1α* siRNA (si*Hif-1α*), or medium alone (Ctrl) for 24 hr with (right) or without (left) $CoCl_2$.
